# Notes and Notices

**Published:** 1883-11

**Authors:** 


### NOTES AND NOTICES.

WANTED.—Dr. T. T. Oliver (3305 Cottage Grove Avenue, Chicago) wishes "a reliable preparation of *Bovilninum* of the highest potency." A graft of this substance will be thankfully received and promptly paid for by the Doctor.

A NEW COLLEGE.—San Francisco is about to have a homœopathic medical college, and judging from selections already made, it is to be really *homœopathic* in its instruction. Like Dundreary's bird, it will have to flock all alone, for no mate can be found. Dr. G. M. Pease has been elected professor of gynecology, and Dr. A. McNeil to the chair of materia medica. Two good selections.

HARD ON I. H. A.—Some people have no reverence, no respect for anything, however sacred, however grand it may be. As an instance of this, we may mention the case of a physician who recently wrote of the I. H. A. as "*an international flock of Swans*" ! !

A TOUGH DIGESTION.—Visitors to the White Mountains have doubtless seen "Jack," who lives on Mt. Willard, at the entrance to the Crawford Notch, go through his frog-eating performance. For those who have not seen it, we may mention that this Jack will, for a small sum of money, swallow six or more live frogs, and seems to relish them! He takes them one by one and drops them, kicking and struggling, down his capacious throat. He draws the line on dead frogs, which he considers indigestible. Jack was shipwrecked once, and spent some months on a desert island, where frogs, worms, etc., were his only diet.

